# A novel Chilean salmon fish backbone-based nanoHydroxyApatite functional biomaterial for potential use in bone tissue engineering

**DOI:** 10.3389/fmed.2024.1330482

**Published:** 2024-05-07

**Authors:** F. Muñoz, Z. S. Haidar, A. Puigdollers, I. Guerra, M. Cristina Padilla, N. Ortega, M. J. García

**Affiliations:** ^1^Facultad de Odontología, Universidad Internacional de Cataluña, Barcelona, Spain; ^2^Laboratorio BioMAT’X R&D&I (HAiDAR I+D+i LAB), Universidad de los Andes, Santiago, Chile; ^3^Centro de Investigación e Innovación Biomédica (CiiB), Universidad de los Andes, Santiago, Chile; ^4^Programa de Doctorado en BioMedicina, Facultad de Medicina, Universidad de los Andes, Santiago, Chile; ^5^Programa de Doctorado en Ciencias Odontológicas, Facultad de Odontología, Universidad de los Andes, Santiago, Chile; ^6^Facultad de Odontología, Universidad de los Andes, Santiago, Chile; ^7^Área de Ortodoncia, Facultat Internacional de Catalunya, Barcelona, Spain; ^8^Laboratorio de Investigación e Ingeniería de Biopolímeros (BiopREL), Universidad de los Andes, Santiago, Chile; ^9^Escuela de Nutrición y Dietética, Facultad de Medicina, Universidad de los Andes, Santiago, Chile

**Keywords:** biomaterials, hydroxyapatite, nanotechnology, osteointegrative, osteoconduction, osteoinduction, salmon bone, bioengineering

## Abstract

**Introduction:**

Given the ensuing increase in bone and periodontal diseases and defects, *de novo* bone repair and/or regeneration strategies are constantly undergoing-development alongside advances in orthopedic, oro-dental and cranio-maxillo-facial technologies and improvements in bio−/nano-materials. Indeed, there is a remarkably growing need for new oro-dental functional biomaterials that can help recreate soft and hard tissues and restore function and aesthetics of teeth/ dentition and surrounding tissues. In bone tissue engineering, HydroxyApatite minerals (HAp), the most stable *CaP*/*Calcium Phosphate* bioceramic and a widely-used material as a bone graft substitute, have been extensively studied for regenerative medicine and dentistry applications, including clinical use. Yet, limitations and challenges owing principally to its bio-mechanical strength, exist and therefore, research and innovation efforts continue to pursue enhancing its bio-effects, particularly at the nano-scale.

**Methods:**

Herein, we report on the physico-chemical properties of a novel nanoHydroxyApatite material obtained from the backbone of Salmon fish (*patent-pending*); an abundant and promising yet under-explored alternative HAp source. Briefly, our nanoS-HAp obtained via a modified and innovative alkaline hydrolysis–calcination process was characterized by X-ray diffraction, electron microscopy, spectroscopy, and a cell viability assay.

**Results and Discussion:**

When compared to control HAp (synthetic, human, bovine or porcine), our nanoS-HAp demonstrated attractive characteristics, a promising biomaterial candidate for use in bone tissue engineering, and beyond.

## 1 Introduction

Bone is a complex yet dynamic structure with an exceptional regenerative potential (1). Consequently, Bone Tissue Engineering (1, 2) continues to be a popular sub-field of study in regenerative medicine and dentistry, also due to the accruing scientific and bone biology knowledge, expertise and technological advances in materials, pharmaceutics, and nano-scaled strategies to achieve *de novo* osseo-regeneration, in simple as well as critical-sized defects (3–5). Indeed, with more than 2 million bone grafting procedures performed annually, around the World, bone tissue is the 2nd most common transplant after blood transfusion (6). Yet, despite critical limitations including supply and quality of host bone (limited bone mass), donor site morbidity, and immunogenicity, autologous and allograft bone grafting materials, respectively, are generally considered the clinical standard-of-care (gold standard) (7). Furthermore, while various synthetic bone graft substitutes are commercially-available, delayed and/or compromised healing remains a significant clinical challenge, today (8). Henceforth, there are still no *ideal* materials available with simultaneously good biocompatibility, biodegradability, porous three-dimensionally, and osseo-conduction, −induction, and-genesis capabilities (9). In this context, the development of *novel* alternatives for safe and effective bone repair, reconstruction, restoration, replacement and/or regeneration is one of the most clinically important long-term goals of R&D&I within the field of mineralized tissues (10). Herein, biomaterials have been established to induce active bio-mineralization owing to their capacity and ability to conduct and induce bone growth; osteo-conduction and-induction, respectively (11, 12). Bone contains about 45–70 wt.% of calcium phosphate (CaP mineralization produces hydroxyapatite in the bone and skeleton formation process), 10 wt.% of water and the remaining is collagen (13). Hydroxyapatite (HAp) minerals, chemically represented as Ca_10_(PO_4_)_6_(OH)_2_, have been extensively studied in the last decades, as is almost identical (close resemblance), to the inorganic portion of the human bone matrix (14). HAp is well-known for its excellent biocompatibility, osteo-conductive and-integrative properties, and thus, is commonly used as a bone graft in orthopedic, oro-dental and cranio-maxillo-facial surgeries (15). In recent years, its potential to apophyse new bone as well as induce the transformation (osteogenic differentiation) of un-differentiated stem cells into osteoblasts (osteo-induction), have been receiving more attention for its application in bone tissue engineering (16, 17), nonetheless, despite being considered the most stable calcium phosphate ceramic material (in terms of temperature, pH and composition of the intra-vascular fluid), critical mechanical (rheological) and safety (biodegradation/resorption over time) issues including low resistance to fracture, fatigue failure, brittleness, high calcium:phosphate ratio and crystallinity, persist. In view of such significant drawbacks or limitations of traditional HAp-based treatments, the search for a novel biomaterial suitable for safe and efficacious bone regeneration, continues. Recent advances in nanoscience and nanotechnology (nanomaterials can be natural, synthetic or composites/hybrids, with a scale of <100 nm) have opened new doors and avenues for designing, formulating, characterizing, optimizing, evaluating, and manufacturing superior nano-scaled bone grafting biomaterials. When compared to traditional porous HAp, nano-scaled HAp has earned considerable attention owing to better bioactivity, superior bone integration ability, physico-chemico-mechanical stability, safety, and biodegradability control, over time. Although HAp can be obtained from animal and human bones, the percentage of the mineral content can vary between species, henceforth, fish bone has been suggested as a good alternative (and sustainable) source. This is especially significant considering that the fishing industry produces billions of tons of fish bone “waste,” annually; a rather major environmental problem. Today, fish bone is processed to be used as feed and a source of calcium for livestock (and protein hydrolysates), however the final products obtained from these wastes may have low value and require higher energy consumption (18, 19). On the other hand, fish sources are presumably much safer than other animal sources, and the wide evolutionary gap between fish and humans suggests a low risk of disease transmission (16). Furthermore, fish by-products are readily-abundant and at a lower cost. Thereby, obtaining HAp from the salmon fish back bone to elaborate new biomaterials can be considered an opportunity and a benefit, helping to reduce the negative environmental impact that the fishing industry waste products present (17, 20). As Chile is the World’s second-largest producer of farmed salmon (estimated at 32% of global production), the waste and pollution problem(s), resource opportunity and potential benefit via innovating a novel inexpensive nano-material as a bio-effective alternative to traditional bone grafts is rather attractive and desirable. When compared to tuna and/or cod fish, salmon can be used to obtain adequate amounts of HAp from their back bones (16, 17, 21). Furthermore, obtaining or producing HAp from the salmon back bone is comparatively an inexpensive process that enables the development of bone grafts with less risk of crossed infection and morbidity, and counteracts any ethical, cultural, or religious issues related to acceptance of other bone grafts, such as xenografts (xenogeneic bone), from bovine or porcine sources. For all of the aforementioned issues and considerations, in this multi-centered collaborative study, we opted to efficiently extract HAp from the back bones of Chilean salmon fish through a modified alkaline hydrolysis – calcination process, conceived in our participating research laboratories, in a simple, reproducible and sustainable manner. The resultant HAp, termed henceforth as nanoS-HAp or SAHA for short (*patent-pending*), was characterized for its physico-chemico-mechanical properties by X-ray diffraction (XRD), scanning electron microscopy (SEM) and energy dispersive X-ray spectroscopy (EDX). Then, the *in vitro* cellular behavior/proliferation and biocompatibility (or cytocompatibility) of the produced nanoS-HAp was tested (17) using a cell viability assay (MTT). Briefly, HAp produced with a nano-scale size, possesses an ultra-structure suitable for a higher absorbability and can provide a higher surface bio-activity favoring biological performance and exhibiting advanced cellular responses (in terms of proliferation and differentiation of osteogenic-related cells for bone regeneration), when compared with traditional HAp. This is a *proof-of-concept* pre-clinical study hypothesizing that nanoS-HAp is a suitable, stable, safe and potentially bio-efficacious bone grafting material alternative to commercially-available natural, synthetic and/or composite hybrid products. It can be expected, in a sequel work (*presently ongoing*), to combine the advantages of our nanoS-HAp with different materials and clinically-relevant conditions, rendered necessary to achieve a better or superior (boosted and accelerated) clinical effect and prognosis, when compared to other formulations and commercially-available products, which often include bone regenerative biomaterials incorporating bio-polymeric, bio-ceramic (inorganic non-metallic materials), calcium phosphates (in powder or cement formats, including α-and β-tricalcium phosphate and tetracalcium phosphate, among others), nano-scaled structures and/or biodegradable matrices with bioactive and easily resorbable fillers; a modern class of bone defect repair, restoration, reconstruction and replacement biomaterials for optimal osseous tissue regeneration, permitting the creation of functional tissue (including soft) substitutes that integrate with the host tissue and restore natural structure and function; a step forward toward developing new or alternative safe and effective (including cost) tissue engineering strategies for routine use in clinical practice, principally, in dentistry, oral surgery, maxilla-facial and cranio-facial surgical interventions.

It is perhaps worth mentioning herein that although many authors have reported on nano-sized or-scaled HAp derived from salmon backbone, our approach deviates via presenting an innovative, simplified, reproducible and sustained method to produce nanoHAp. This process results in an ultra-structure that promotes higher absorbability and greater surface bioactivity. Furthermore, our study provides a pre-clinical proof-of-concept suggesting that nanoS-HAp is a safe/biocompatible, stable, and potentially more effective alternative bone grafting material when compared to other commercially-available products. Our ongoing research aims at combining the advantages of nanoS-HAp with different clinically-relevant materials and conditions, with the goal of achieving improved or superior clinical effects compared to current formulations and bio-products available on the market.

## 2 Materials and methods

### 2.1 Extraction

Salmon fish bone waste was obtained from a local/national industrial fish farm and the skeleton cut into small pieces with muscle and fat removed, manually. Skeleton pieces were then washed with distilled water at 95°C and stirred constantly at 200 rpm for 1 h to re-move impurities such as lipids and proteins. Next, the residual water and tissues were all removed, and the samples left to dry. The remaining bone was cleaned with distilled water 3 times for 2 min under 200 rpm stirring. Finally, the treated bones were added to a HCl solution (1:6 ratio, set at 4°C) for 15 h under 150 rpm stirring.

### 2.2 Synthesis

HA extraction from the salmon bones, has been previously described in the literature (17). Herein, modifications were made to the protocol, mainly for viability reasons. Briefly, 100 grams of salmon skeleton were treated through an alkaline hydrolysis – calcination process to obtain HA. The skeleton was dried out at 60°C for 12 h prior to calcination. The sample was then crushed in a Foss KN 95 Knifetec^®^ mill and subjected to a calcination process at 650°C in a heated muffle for 5 h, then stored in a laboratory freezer at-80°C, essentially to prevent moisture absorption by the sample(s). Finally, a total of 7.34 grams of HA was obtained and then stored to be further characterized and used afterwards.

### 2.3 Characterization

#### 2.3.1 Biological test for *in vitro* cytocompatibility: MTT and WST-1 spectrophotometric assays

To determine the cell proliferation and differentiation capacity of the hydroxyapatite samples obtained from the salmon bone, a cell viability colorimetric assay/test (MTT) was performed evaluating the cell metabolic activity of the cultures (17). A fresh stock of each type of sub-stratum [salmon (SAHA), human (HHA), bovine (BHA), porcine (PHA) and synthetic (SHA)] was prepared before each *in vitro* test. The concentration of each stock was set at 5 mg/mL. This solution was then sonicated for 10 min. All stocks were autoclaved and then made up to their total volume using sterile distilled water and adding DMSO (Dimethylsulfoxide, CH₃SOCH₃) at 0.1% v/v. Dilutions were prepared with final concentrations of substrates, as follows: 0; 50; 100; 150; 200; 250 and 300 ug/mL, prepared in cell culture medium. The test was performed on 2 different cell lines/cultures, as is briefly described below:

- C2C12 cells (myoblasts: immortalized mouse myoblast cell line) were cultured in DMEM culture medium (Dulbecco’s Modified Eagle’s Medium) supplemented with 10% FBS (fetal bovine serum), 2 mM glutamine and antibiotics (100 U/mL penicillin and 100 ug/mL streptomycin).
- MEF cells (fibroblasts: murine embryonic fibroblast cell line) were cultured in DMEM culture medium supplemented with 15% FBS, 2 mM glutamine, 1 mM pyruvate and antibiotics (100 U/mL penicillin and 100 ug/mL streptomycin).

The cells were cultured with 5% CO_2_ and incubated at 37°C. The viable cell concentration was then determined using spectrophotometry using the commercial WST-1 Cell Proliferation kit (Roche). The relative cell viability (percentage %) was determined after 24 h of being in contact with the substrate:


$$\mathrm{Viability}\%=\frac{\begin{array}{l} \mathrm{viable}\;\mathrm{cells}\;\mathrm{after}\;24\;\mathrm{hrs}\;\mathrm{of}\;\\ \mathrm{contact}\;\mathrm{with}\;\mathrm{substrate}\; \end{array}}{\begin{array}{l} \mathrm{viable}\;\mathrm{cells}\;\mathrm{after}\;24\;\mathrm{hrs}\;\\ \mathrm{without}\;\mathrm{substrate} \end{array}}\;x100$$

All experiments were done in triplicate, and results are reported as mean ± standard error.

#### 2.3.2 Physical tests for structural properties: XRD, SEM, EDS, and RAMAN testing

X-ray powder diffraction (XRD) was performed to analyze the anatomical and molecular structure of the obtained/produced HA crystals (16, 17, 20–25). XRD analysis was performed using an X-ray diffractometer (Phillips X’Pert Pro, United Kingdom) equipped with a CuKα radiation source set at a wavelength of 1,5,409 Å. Studies were run at a current of 30 mA and an accelerating voltage of 40 Kv, over the 2θ diffraction angle range of 2° to 80° using a step size of 0.02°. The patterns obtained were analyzed using the Origin pro 2019b software (OriginLab Corp., United States). Further, scanning electron microscopy (SEM) and energy dispersive X-ray spectroscopy (EDS) were used to evaluate the Ca/P ratio of the samples. A Carl Zeiss scanning electron microscope (EVO MA 10, Oberkochen, Germany) equipped with energy dispersive spectroscopy (EDS) was used to analyze local chemical composition of samples. Finally, the nano-milled powder was then collected and centrifuged to separate n-HAp particles followed by drying, another milling/grounding round, and samples were labeled for further use and analysis. Raman scattering spectroscopy measurements of the HA obtained by mechanical alloying were obtained. Briefly, the Raman spectra were measured using a triple monochromator micro-Raman spectrometer, equipped with a charged coupled device (CCD) detector, and using the 4,880 Å exciting line of the Ar-laser. All the aforementioned assessments were performed at the CiiB.

#### 2.3.3 Control group(s)

**Figure 1:**
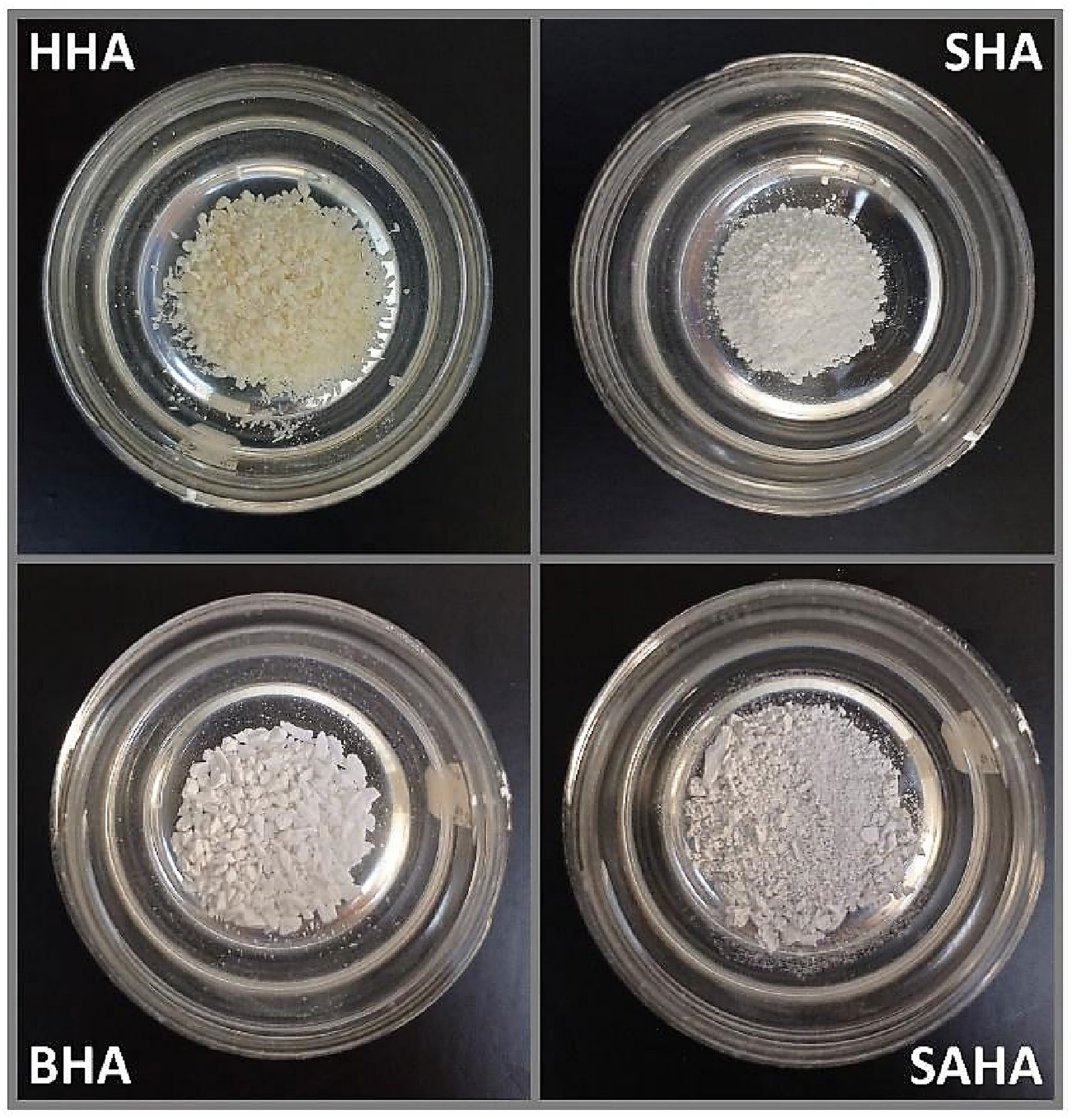
Prepared control and experimental biomaterial samples.

For this *in vitro* study, controls included hydroxyapatite powder samples of distinct origins (Figure 1): hydroxyapatite of synthetic origin (Sigma Aldrich^®^, Germany), xenograft of porcine origin (The Graft, Puro Biologics, United States), human allograft (OraGraft Mineralized Cortical, Lifenet Health, United States) and bovine xenograft (Mineross Cortical X, Biohorizons, United States), for a most-possible comparative analysis.

## 3 Results

**Figure 2:**
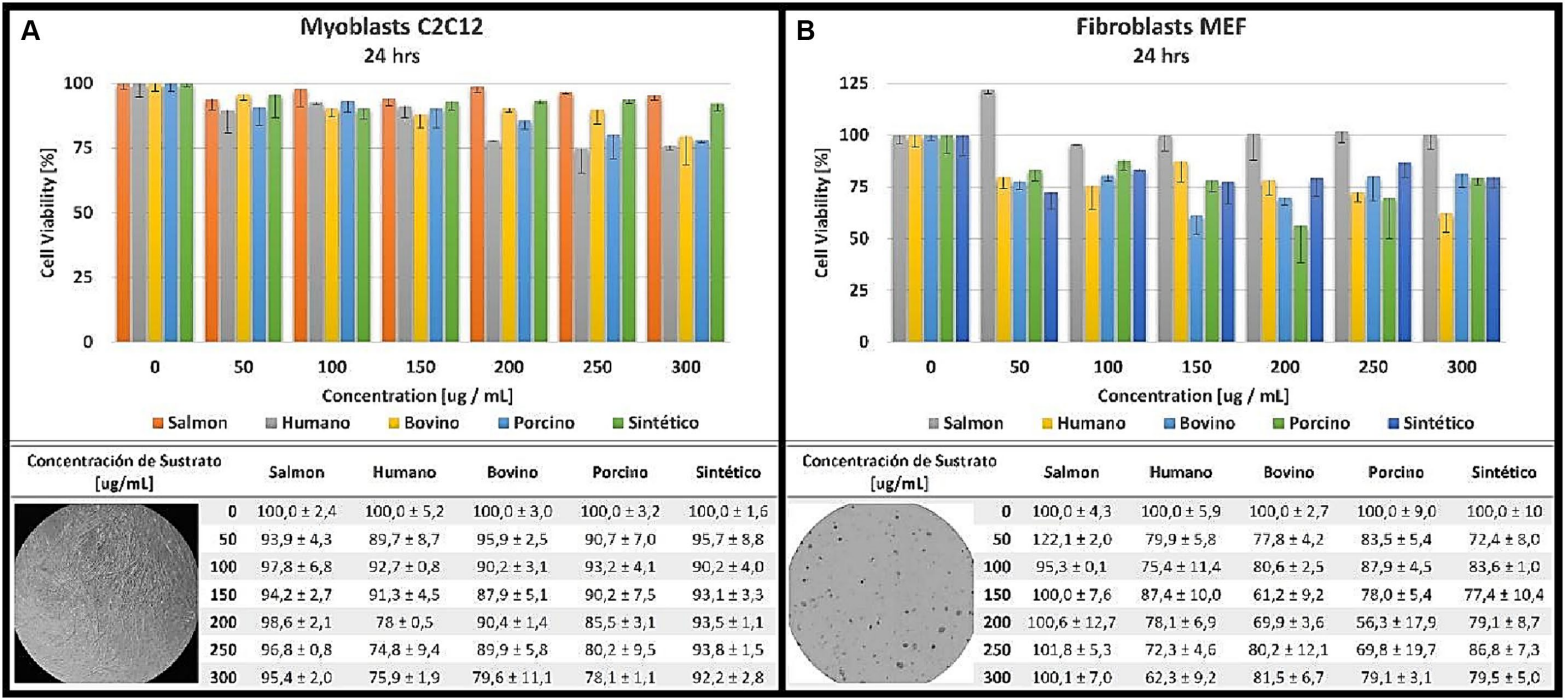
Cytotoxicity analyzed at different concentrations for 24 h in myoblasts **(A)** and fibroblasts **(B)**.

As displayed in Figure 1, we were successful in obtaining/producing nanoS-HAp/SAHA, in-House, with comparative characteristics to commercially-available Ca/P-based variations and at usable quantities. The determination of the general cellular activity is important to verify that the material obtained from salmon bone can be used as a possible bone replacement material, safely. Findings of the *in vitro* cyto-viability/−toxicity test are demonstrated in Figures 2A,B. The 5 substrates (SHA, BHA, PHA, HHA and SAHA) were evaluated at different concentrations over a period of 24 h, with both, C2C12 cells (myoblasts) and in MEF cells (fibroblasts). The results obtained for the myoblast cell cultures indicate that the substrate corresponding to salmon bone *significantly* promoted the cell viability of the myoblasts at a concentration of 100 [ug/mL] and more, showing the highest cell viability for this substrate at a concentration of 200 [ug/mL] similar to previous reports in the literature (26–28). On the other hand, the substrate corresponding to HHA showed cell viability below 80% at concentrations of 200 [ug / mL] and more. A yield over 90% viability was demonstrated for porcine, bovine, and synthetic samples at concentrations of 100 [ug/mL]. The results obtained herein for the cytotoxicity test in fibroblasts turned out to be highly-comparable to those obtained in myoblasts. The highest cell viability was reached in the substrate corresponding to SAHA at a concentration of 50 [ug/mL]. It was evidenced that the porcine substrate at concentrations of 150 [ug/mL] and more, does not have obvious or clear promoter effects on cellular viability and proliferation during the culture period. Henceforth, the substrate based on our salmon bone corresponds to the substrate with the highest percentage of viability and proliferation in all concentrations, throughout the monitored period.

**Figure 3:**
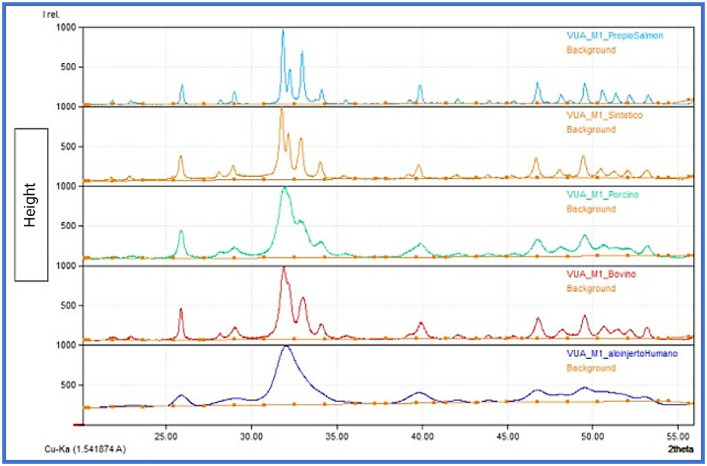
XRD graphs obtained, comparison of the samples.

Then, the crystallite size was studied using XRD and the size was further analyzed using the Scherrer equation. Sizes ranged from 8.8 nm to 60.38 nm (Figure 3; Table 1), with the smallest crystallite size being the HHA at 8.8 nm and the largest being 60.38 nm for the SAHA sample, similar to the previously reported literature (26–28). Likewise, the crystallite size found in the synthetic sample corresponds to 35.29 nm, indeed, similar to that reported by Venkatesan et al. (1, 12), further verifying our experimental protocol.

**Table 1:** Crystallite size obtained by XRD for SHA, HHA, BHA, PHA, and SAHA samples.

Sample	Crystallite size (nm)
Synthetic HA (SHA)	35.29
Human HA (HHA)	8.8
Bovine HA (BHA)	22.26
Porcine HA (PHA)	12.25
Salmon HA (SAHA)	60.38

HA, hydroxyapatite; SHA, synthetic HA; HHA, human HHA; BHA, bovine HA; PHA, porcine HA; SAHA, salmon HA.

**Figure 4:**
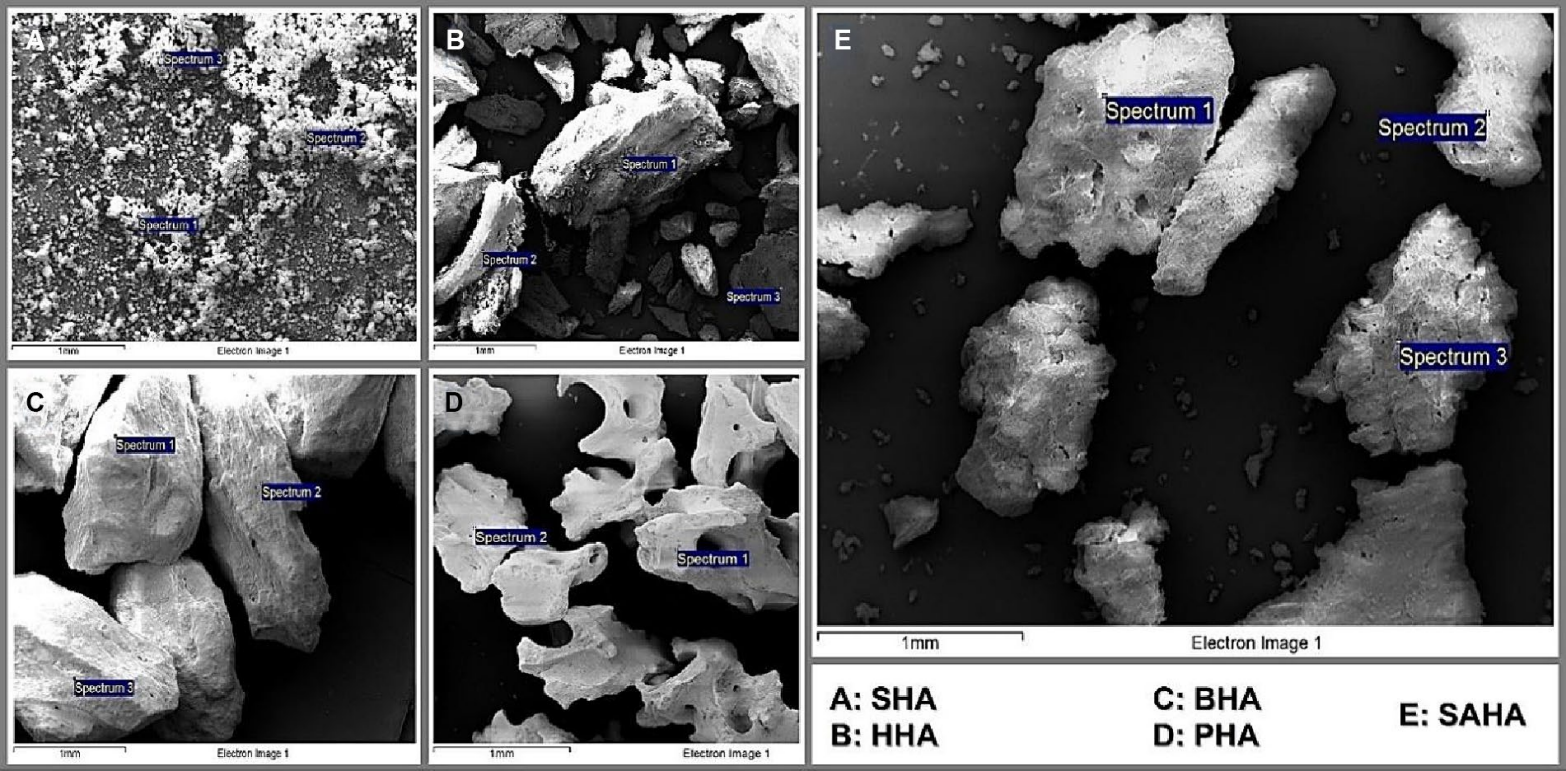
SEM micrographs of SAHA **(A)**, SHA **(B)**, BHA **(C)**, HHA **(D)**, and PHA **(E)** samples.

**Figure 5:**
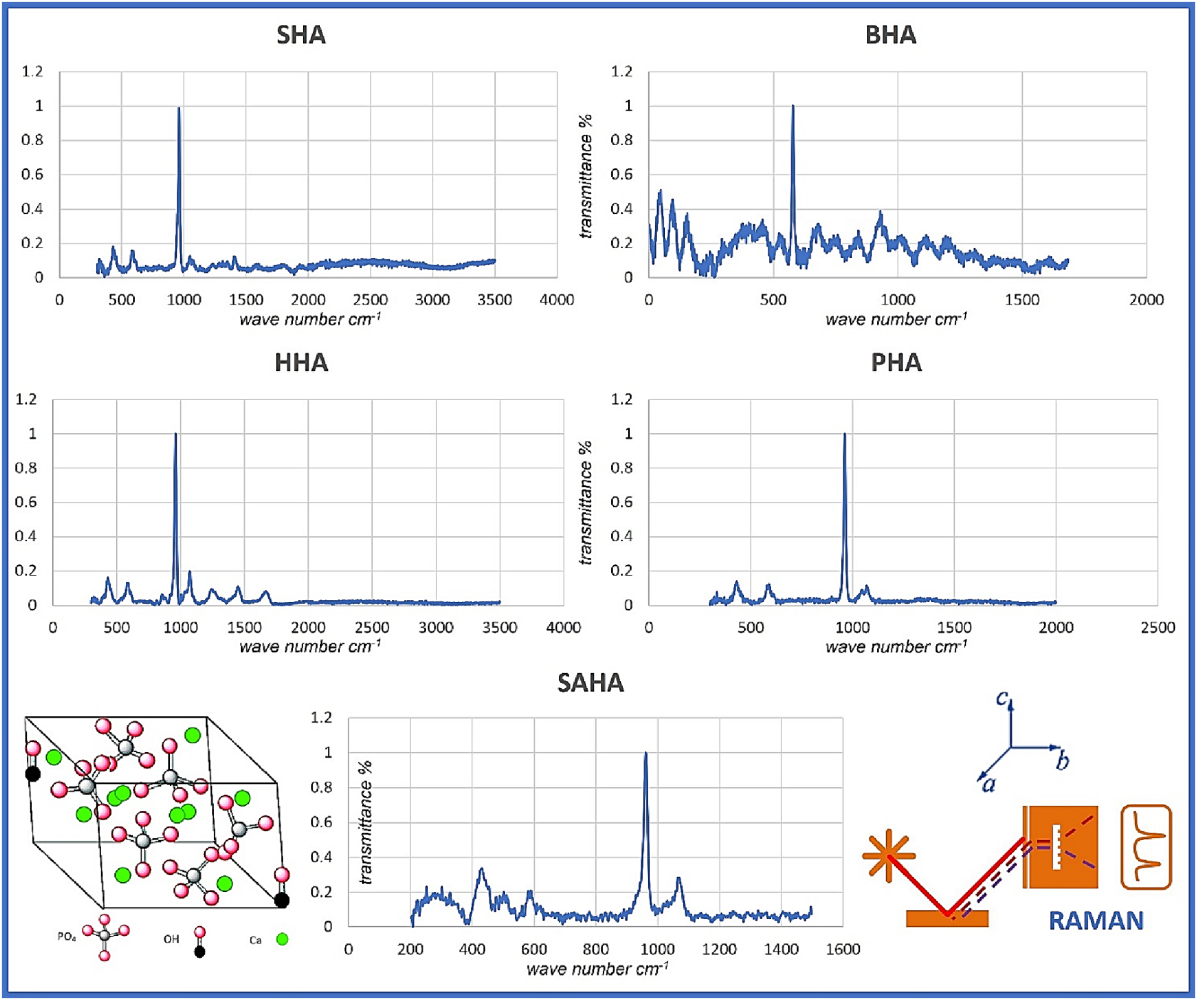
Raman spectroscopy/spectra of all samples employed in the study.

Using SEM/EDS analysis, the Ca/P ratio for SHA, HHA, BHA, PHA and SAHA samples was obtained/determined (Figure 4; Table 2). The lowest values obtained were for SHA and BHA with 1.94 and 1.98 respectively, similar to what is often presented in the literature for the stoichiometric value of HA (at 1.67). However, all the samples displayed values higher than commonly reported in the literature. Finally, samples were placed on a glass slide and were focused by the Ar-laser beam prior to acquiring the spectrum. Spectra were obtained for a small area and averaged for interpretation. Characteristic peaks for each sample were obtained depicting distinct peaks for the SAHA. Using Raman spectroscopy analysis, the peaks corresponding to each functional group were carefully identified and analyzed. Herein, peaks at 961 were consistently observed across all samples, indicating the presence of the phosphate (PO_4_) groups (Figure 5). Additionally, the yielded peaks at 1449 and 608, corresponding to (PO_4_) and (CO_3_) respectively, were detected in the HHA sample, suggesting the presence of the classical or typical hydroxyapatite structure (1,5,12), as to be expected. Conversely, and interestingly, no peaks indicative of CO_3_ and OH groups were found in the salmon bone samples.

**Table 2:** Ca/P ratio obtained by SEM/EDS for SHA, HHA, BHA, PHA, and SAHA samples.

Sample	Ca/P RATIO
Synthetic HA (SHA)	1.94
Human HA (HHA)	5.85
Bovine HA (BHA)	1.98
Porcine HA (PHA)	3.13
Salmon HA (SAHA)	2.61

HA, hydroxyapatite; SHA, synthetic HA; HHA, human HHA; BHA, bovine HA; PHA, porcine HA; SAHA, salmon HA.

## 4 Discussion

Distinguishing between osteoconductivity and osteoinductivity within the realm of biomaterials, such as the novel and *patent-pending* Chilean salmon fishbone-based hydroxyapatite material investigated in this study, holds significant implications, particularly in light of our presented *preliminary*/*proof-of-concept* research findings. To recap, osteoconduction, characterizes the ability of a material to passively facilitate the infiltration of host capillaries, perivascular tissue, and mesenchymal stem cells. Our assessment of cellular viability performed/conducted in two distinct cell lines, in conjunction with advanced characterization techniques including SEM, XRD, Raman spectroscopy, and EDS, revealed that our SAHA possess structural similarities to cancellous bone. This observation has important implications for their application in bone tissue engineering. Notably, osteoconductive materials, such as the one we have examined, offer an alternative biomaterial/matrix candidate upon which osteoblasts and osteoclasts can firmly anchor, migrate, proliferate, and collaborate. Our findings thus underscore the potential of these materials to actively foster bone cell attachment, growth, and repair. Furthermore, our findings suggest that the extracted Chilean salmon fishbone-based hydroxyapatite material also exhibits qualities of osteoinductivity. Osteoinductivity encompasses the capacity of a biomaterial to actively induce the differentiation of MSCs into osteoblasts, thereby stimulating bone formation. Our analysis, including the assessment of cellular viability and the advanced techniques employed, indicates the potential of this biomaterial to potentially serve as an osteoinductive agent and/or matrix. Henceforth, this study, supported by the employed suite of in-House available techniques, extends our understanding of the intricate interplay between biomaterials, cellular viability, and physico-chemical characteristics in the context of tissue engineering, opening new avenues for innovative clinical uses.

The incorporation of various hydroxyapatite variants has been a subject of global research, extensively documented in existing literature. The employment of this mineral type in bone tissue engineering offers a promising prospect for mending both straightforward and intricate defects. Hydroxyapatite, a calcium phosphate mineral naturally occurring in bone, has garnered significant attention. Notably, fish bones, hitherto regarded as waste material, have been unveiled as a valuable and potentially cost-effective, sustainable source of hydroxyapatite with promising applications in the realm of bone repair.

The conducted cyto-viability/−toxicity analysis has yielded promising results, demonstrating the potential of our biomaterial. We conducted tests on mouse fibroblast and myoblast cells, chosen in this study for their capacity to differentiate and proliferate into pre−/osteoblasts, a pivotal cell type in bone formation. Notably, the introduction of bone morphogenic protein 2 (recombinant human BMP-2) induced a notable shift in the differentiation pathway, a development that holds considerable promise for future investigations focused on bone formation ability and cellular viability within osteoblasts (29–33). In our imminent formulation, osteogenic proteins can be incorporated to boost/accelerate function.

The evaluation of cell viability, expressed as a percentage, exceeded 90% for our Chilean salmon fish bone-based substrates in myoblast culture at all concentrations after 24 h. Moreover, the results were even more encouraging when culturing the murine fibroblasts on those substrates, where a remarkable 122% increase in cell viability was observed at a *low* concentration of 50 [μg/mL], further highlighting and/or confirming their contributory potential, when compared to the other commercially-available materials. These outcomes also underscore the non-toxic nature of our SAHA powder across all studied concentrations. Importantly, it appears that physical properties of hydroxyapatite, including factors such as density, crystallization, and elemental composition, significantly influence cell viability (1, 12). Notably, the presence of functional groups, like CO^3^-2_-x_, has been associated with enhanced cell adhesion and proliferation, potentially promoting the formation of mineralized tissue (34). To gain deeper insights into the acquired hydroxyapatite, *in vivo* preclinical studies are warranted and ongoing.

Our research also indicated and confirmed that hydroxyapatite derived from natural sources is likely to contain the essential mineral ions that contribute to improved cell adhesion, proliferation, differentiation, and the formation of mineralized tissue. Intriguingly, our XRD analysis revealed variations in crystallite size among all samples, yet all exhibited acceptable levels of cell proliferation, suggesting that crystallite size may not be the sole determinant for cell proliferation (35). Herein, additional studies focusing on surface topography and its relevance in cell adhesion and proliferation are considered. Nonetheless, our performed SEM/EDS analysis further revealed that all samples exhibited higher values of the Ca/P ratio when compared to stoichiometric HA (hydroxyapatite) with a ratio of 1.67, similar to several previous literature reports. It is noteworthy that elevated Ca/P ratios can be indicative of reduced carbon content within the material. Intriguingly, a heightened presence of carbon has previously been correlated with a phenomenon known as *selective* protein adsorption, suggesting its potential role in fostering specific protein interactions that, in turn, facilitate enhanced cellular adhesion to the substrate (35–40). This phenomenon of selective protein adsorption may provide valuable insights into the complex interplay between material composition and cellular response. Our ongoing *in vivo* study is designed to further explore the underlying signaling pathway(s).

## 5 Conclusion

Today, bone tissue engineering continues to stand as an exciting frontier in medicine and therapy, brimming with opportunities for research, development, and innovation. Its primary objective is the restoration, repair, reconstruction, replacement, and regeneration of bone structure and function following injury, disease, or surgical intervention. In light of the relentless progress in the fields of biomaterials, polymers, nanotechnology, tissue engineering, and regenerative medicine, scientists and clinicians continue to explore novel approaches that promise to enhance the healing process while devising novel bone substitutes that satisfactorily mimic the intricacies of natural soft and hard tissue(s). Amid this landscape of innovation, fish and fish bone waste emerge as a noteworthy player due to its significant content of calcium phosphate. Salmon bone, in particular, has emerged as a sustainable and potentially transformative source of hydroxyapatite. This remarkable substance possesses the unique capacity to foster the growth and differentiation of bone cells, positioning it as a promising and viable alternative biomaterial with broad applications in both, biomedical and dental tissue engineering. Our preliminary *proof-of-concept* work presented herein underscores the similarity between salmon backbone particulate and bone hydroxyapatite mineral, both in biological and physico-structural properties. This likeness is not only encouraging yet also suggests that this biomaterial is non-cytotoxic, physically stable, and amenable to modulation and customization/personalization. As a testament to its potential, a patent application has been submitted, marking the first step in the journey toward realizing its full utility and translation to the clinic and the end-user. However, it is deemed vital to acknowledge that challenges and limitations continue to persist in the utilization of fish waste-derived hydroxyapatite. Standardized extraction methods must be established, and the absence of contaminants assured. Henceforth, our ongoing studies delve into biocompatibility and bio-degradation characterization alongside the histopathological and immune-histochemical profile of SAHA, *in vivo*, in order to offer a more valuable comparative perspective on the rate of *de novo* bone tissue formation. In this context, the horizon of tissue engineering holds the promise of not only innovative biomaterials but also a deeper understanding of the complex interplay between materials and biology, inspiring the ongoing quest for medical therapy advancements that will revolutionize tissue repair and regeneration.

## Data availability statement

The raw data supporting the conclusions of this article will be made available by the authors, without undue reservation.

## Ethics statement

Ethical approval was not required for the studies on animals in accordance with the local legislation and institutional requirements because only commercially available established cell lines were used.

## Author contributions

FM: Conceptualization, Data curation, Formal analysis, Funding acquisition, Investigation, Project administration, Resources, Writing – original draft. ZH: Conceptualization, Data curation, Formal analysis, Funding acquisition, Methodology, Project administration, Resources, Supervision, Validation, Writing – original draft, Writing – review & editing. AP: Investigation, Writing – original draft. IG: Investigation, Writing – original draft. MP: Investigation, Writing – original draft. NO: Investigation, Writing – review & editing. MG: Investigation, Writing – original draft.
